# Multifunctional Cu_2_SnS_3_ Nanoparticles with Enhanced Photocatalytic Dye Degradation and Antibacterial Activity

**DOI:** 10.3390/ma15093126

**Published:** 2022-04-26

**Authors:** Harshad D. Shelke, Archana R. Machale, Avinash A. Survase, Habib M. Pathan, Chandrakant D. Lokhande, Abhishek C. Lokhande, Shoyebmohamad F. Shaikh, Abu ul Hassan S. Rana, Marimuthu Palaniswami

**Affiliations:** 1 Advanced Physics Laboratory, Department of Physics, Savitribai Phule Pune University, Pune 411007, Maharashtra, India; harshadshelke4@gmail.com (H.D.S.); pathan@physics.unipune.ac.in (H.M.P.); 2 Solid State Physics Laboratory, Department of Physics, Yashwantrao Chavan Institute of Science, Satara 415001, Maharashtra, India; arch.machale@rediffmail.com; 3 Rayat Institute of Research and Development Center, Satara 415001, Maharashtra, India; surwasedada09@gmail.com; 4 Centre for Interdisciplinary Research, D. Y. Patil Educational Society, Kolhapur 416006, Maharashtra, India; 5 Department of Physics, Khalifa University of Science and Technology, Abu Dhabi P.O. Box 127788, United Arab Emirates; abhi4502@gmail.com; 6 Department of Chemistry, College of Science, King Saud University, P.O. Box 2455, Riyadh 11451, Saudi Arabia; sshaikh1@ksu.edu.sa; 7 Department of Electrical and Electronic Engineering, The University of Melbourne, Parkville, VIC 3010, Australia; palani@unimelb.edu.au

**Keywords:** antimicrobial activity, dye degradation, methylene blue, photocatalytic movement, pathogenic strain, nanoparticles, hydrothermal

## Abstract

We present a simplistic, ultrafast, and facile hydrothermal deposition of ternary Cu_2_SnS_3_ nanoparticles (CTS NPs). The fabricated CTS NPs show superior antimicrobial and photocatalytic activities. In the presence of UV-Visible illumination, methylene blue (MB) dye was studied for photocatalytic dye degradation activity of CTS NPs. Excellent efficiency is shown by incorporating CTS NPs to degrade MB dye. There is a ~95% decrease in the absorbance peak of the dye solution within 120 min. Similarly, CTS NPs tested against three bacterial strains, i.e., *B. subtilis*, *S. aureus*, *P. vulgaris*, and one fungal strain *C. albicans*, defining the lowest inhibitory concentration and zone of inhibition, revealed greater antimicrobial activity. Hence, it is concluded that the CTS NPs are photocatalytically and antimicrobially active and have potential in biomedicine.

## 1. Introduction

The textile industry zone is considered the prevalent wastewater and overwhelming dye sector. Effluents unconstrained from such industries contain various pollutants that are hazardous to human health and the environment. Voluminous processing, such as printing and dying the cloths in textiles, also consumes large amounts of water and dyes. They discharge large quantities of wastewater, which contain significant amounts of dyes harmful to the ecosystem and human beings [1]. Dye is a chemical, colored substance used to form chemical bonding with the substrate. Dyes are the organic compounds almost universally employed in industries such as paints, food, plastics, paper, pulp, textile, pharmaceutical, and leather [2,3]. Dyes are categorized into diverse types according to their structure, function, or both. Acidic, basic, direct, disperse, reactive, cationic, anionic, etc., are the categories of dyes. Due to the colored nature of pigments and dyes, they absorb only specific wavelengths of visible light. Dyes are water-soluble, and pigments are water-insoluble. Lake pigments can be produced by rendering some dyes with salt. These tints are mutagenic, allergic, and carcinogenic and are therefore hazardous to flora and fauna of the biota. Dyes are active in an aqueous media, which helps improve the fastness of the dye on the substance [4]. 

Methylene blue (MB), also known as methyl thioninium chloride, is one of the foremost pollutants. It is an artificial chemical compound with a pungent structure and is a cationic organic dye. It is carcinogenic and toxic to living species. It irritates the eye and skin and affects the respiratory system [5,6]. MB-contaminated drinking water has been recognized as harmful to humans and animals and results in subcutaneous tissue-borne sarcoma [7]. The dye affects aquatic life, particularly plants, as it obstructs light diffusion and hence shrinks natural purification and photocatalysis [8]. Therefore, it is required to confiscate the dye before discharging it into freshwater. Before wastewater treatment, some conventional physical, biological, and chemical methods are responsible for environmental degradation [9]. Hence, radical oxidation processes have attracted copious attention from researchers to deal with wastewater purification. Radical oxidation processes have many advantages, as almost all dyes can be oxidized. They exhibit non-selectivity and are of the swift process [8]. Fenton reactions [10], ozonation [11], photolysis [12], photocatalysis [13], sonolysis [14], and sonocatalysis [15] are techniques that are incorporated in radical oxidation processes [8]. Amid these, photocatalysis has attracted the attention of researchers because it is a green, unique, effective, and simple technology offering ecological and nontoxic end products [9]. Photocatalysis is defined as the catalysis-driven acceleration of a light-induced reaction, and it is divided into two types: (1) homogeneous photocatalysis and (2) heterogeneous photocatalysis [16]. The heterogeneous catalysis process deals with the degradation of many organic pollutants present in wastewater [17,18], which offers numerous advantages, such as being relatively inexpensive, as well as its whole degradation into biodegradable and non-toxic end products. It requires only mild temperature and pressure conditions [16].

Organic poisonous waste and some other active toxins present in wastewater are responsible for diminishing the quality of water. The toxins are nothing but fragments of microbes, which are responsible for increasing pathogenic content in water [19,20,21,22]. It is the ultimate requirement of a photocatalyst to consume the inhabitation ability of microbial pollutants. The nanoparticles have antimicrobial properties against both gram-negative and gram-positive bacteria, and they must have size, surface charge, morphology, thermal, optical, and photoactive properties [19,20]. Various bacteria show flexible susceptibility towards NPs [21,22]. Bacterial resistance against antimicrobial drugs is caused due to dissipated use of antibiotics, therefore, to overcome this resistance problem, a well-organized antimicrobial agent is required [23]. Many semiconductor materials, such as metal oxides, sulfides, and selenides, are used as heterogeneous semiconductor photocatalysts and antimicrobial-resistant materials, with band gap energies ranging from 1.0 to 3.8 eV [24,25,26,27,28]. Metal sulfides and metal selenides have drawbacks, such as stability, photoanodic corrosion, and toxicity [29]. Metal oxides are mainly used as photocatalysts and antimicrobial agents. Their favorable photocatalytic properties have attracted considerable interest, i.e., high catalytic activity, nontoxicity, physiochemical stability, and electronic, thermal, and chemical properties. [16]. However, unfortunately, metal oxide semiconductors also have a central issue with wide band-gap. Comprehensive band-gap materials are dynamic in an ultraviolet spectral range of the solar spectrum and are inefficaciously utilized in practice due to poor photocatalytic activity in visible light [30,31]. Metal oxides also show low photo quantum efficiency because of the small lifetimes of the charge carriers [32,33]. Since it is desirable to use sunlight effectively for energy production, water purification, and environmental protection, low band-gap semiconducting materials play a vital role as photocatalysts and antibacterial agents [34,35].

Ternary Cu_2_SnS_3_ (CTS) material has a tunable band-gap, making it a good candidate for photocatalysis using visible light (sunlight) and antimicrobial applications. The appropriate band-gap, abundant and low cost of Cu, Sn, and chalcogen elements (S and Se) have also made them strong candidates for various applications. The conductivity of CTS is a p-type showing bulk band-gap in the range of 0.9 and 1.50 eV, and the coefficient of optical absorption of materials is 10^−4^ cm^−1^. Therefore, CTS is an appropriate material for use as a photocatalyst and antimicrobial agent. Many researchers have tried various chemical and physical approaches to synthesizing ternary CTS material. [36,37]. The present study shows that CTS nanoparticles are synthesized using a simple, economical, and eco-friendly hydrothermal route and are characterized structurally and optically. Degradation of MB dye was performed using synthesized nanoparticles in visible light illumination and antimicrobial activity in the case of three bacterial strains, i.e., *S. aureus*, *P. vulgaris*, *B. subtilis*, and one fungal strain, *C. albicans*, was also tested.

## 2. Materials and Methods

### 2.1. Synthesis of CTS Nanoparticles

**Figure 1:**
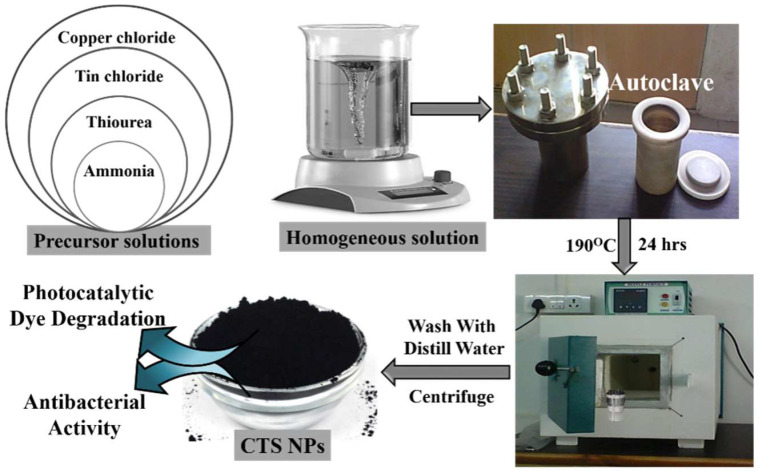
Schematic representation of hydrothermal deposition method of CTS NPs and its applications.

All of the chemicals utilized in the synthesis of CTS NPs are of analytical grade. All chemicals were purchased from Sigma Aldrich, Saint Louis, MO, USA. Briefly, 0.8 mmol of tin (II) chloride-dehydrate (Sncl_2_·2H_2_O), 1 mmol of copper (I) chloride (CuCl·2H_2_O), and 4 mmol of thiourea (CH_4_N_2_S) were individually dissolved in 30 mL of deionized water with constant stirring for 10 min. First, the CuCl_2_·2H_2_O solution was mixed into the SnCl_2_·2H_2_O solution and stirred for 15 min. Then, CH_4_N_2_S solution was slowly mixed with the above mixture with gentle stirring. A white precipitate was observed after adding CH_4_N_2_S. Then, the pH of the solution reached approximately 9.0 by using a liquor ammonia solution. To synthesize CTS NPs, the hydrothermal method was carried out in 100 mL Teflon-lined stainless-steel autoclave at a temperature of 190 °C for ~24 h (heating rate 5 °C/min). To increase the crystallinity of CTS NPs, the collected powder was washed a few times with distilled water, and the obtained precipitate was dried at 80 °C in a vacuum oven for 2 h (Figure 1).

### 2.2. Characterization

A UV-visible spectroscopy analysis was used to measure the optical transparency of CTS NPs (V-530, Jasco, Tokyo, Japan). The band-gap was calculated by diffuse reflectance spectroscopy through Kubelka-Munk (K-M) function. SEM (JEOL JSM-6390, Tokyo, Japan) digital silhouettes were used to observe the surface morphology of the CTS NPs. The X-ray diffraction (XRD) spectrum with a Cu-radiation tube (K_α_ of λ = 1.54 Å) was used to investigate the crystal phase (BRUKER AXS D8 Advanced model, Billerica, MA, USA). The elemental composition was determined using an X-ray photoelectron spectroscopy profile obtained with (VG Multilab 2000 instrument, Thermo VG Scientific, Oxford, UK). Furthermore, FTIR spectroscopy analysis was performed using a Jasco FT-IR/4100 spectrometer. The specific surface area and pore size distribution of CTS powder are studied by BET and Barrett–Joyner–Halenda (BJH) analysis using the Quantachrome Instruments v11.02 model.

### 2.3. Photocatalytic Activity Measurement

To investigate the catalytic activity of the CTS NPs, photocatalytic degradation of MB dye was carried out under sunlight illumination. A 50 mL solution of MB dye (concentration: 1 × 10^−5^ M) was used as an organic impurity. The beaker holding MB dye solution amidst the CTS photocatalyst was laid on the magnetic stirrer, and this entire assemblage was positioned under sunlight illumination. We used a visible light source, an LED light source (6 W), combining a 585-nm LED lamp and a 613-nm LED lamp for the photocatalysis analysis. 

### 2.4. Antimicrobial Activity Measurement

The sterile saline water was used to grow microbial cultures in inoculums. For the growth of bacteria, the nutrient agar plates were used as a medium. The development of *C. albicans* was spread on sterile sabouraud agar plates; likewise, the growth of *S. aureus*, *B.* *subtilis*, and *P. vulgaris* cultures was spread on clean nutrient agar plates. Using a micropipette, CTS NPs were dispersed in dimethyl sulfoxide (DMSO) and sterile distilled water. To observe antibacterial activity, plates were kept incubated at 37 °C for 24 h.

## 3. Results

### 3.1. Structural Elucidation

**Figure 2:**
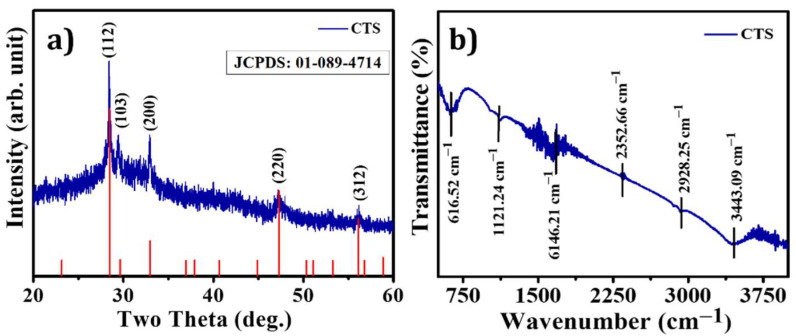
(**a**) X-ray diffraction pattern, (**b**) FTIR spectrum of CTS NPs.

The possible growth mechanism of CTS powder was proposed by using XRD, FTIR, and XPS techniques. During hydrothermal synthesis in airtight vessels, chemical reactions occur when the solution is heated at elevated temperatures. The growth mechanism involves coagulation, recrystallization, and nucleation depending on the temperature and reaction time [38]. To confirm that CTS NPs had indeed been formed and had the expected composition, structure, and phase, and XRD analysis was performed. The powder XRD pattern of CTS NPs is shown in Figure 2a. The XRD peaks appearing at 2θ = 28.56°, 29.62°, 33.06°, 47.40°, and 56.29° are attributed to the (112), (103), (200), (220), and (312) planes, respectively, and the planes of the tetragonal structure matched well with those of kesterite-type CTS [39,40]. The three peaks at 2θ = 28.56°, 47.40°, and 56.29° are assigned to the (112), (220), and (312) lattice planes of a tetragonal structure (JCPDS: 01-089-4714). No impurity peaks and secondary phases occurred in the XRD analysis of binary sulfides such as Cu_2_S and SnS_2_ materials. The Scherrer formula was applied to calculate the average particle size, which was 19 nm, cantered on the full width at half maximum of the (112) diffraction peak. FTIR spectroscopy was used to identify the covering ligands on the surface of CTS NPs, as shown in Figure 2b. The prepared CTS NPs were illuminated to the IR radiation in the form of a pallet. A pallet fabrication was conducted by mixing a small amount of the formed powder into potassium bromide (KBr), and all mixture was ground homogeneously for the construction of the sample into the KBr base. Consequently, the mixture was hard-pressed in a hydraulic press instrument applying a pressure up to ~7 to 8 tons. The spectrum was obtained from 500 cm^−1^ to 4000 cm^−1^ with the resolution of 2 cm^−1^. It is evident in Figure 2b that the vibrations reflected between 500 and 750 cm^−1^ correspond to Cu-S, Sn-S, Sn (IV)-O, and Sn (II)-O bonds present in the synthesized material [41]. The small peak in the spectra, which occurred at 2341.98 cm^−1^, confirms the typical mode of Sn-S bond vibrations found in CTS NPs such as SnS and SnS_2_ [42,43]. The asymmetric stretching of the carbonyl (C=O) causes the absorption band at 1116.70 cm^−1^. The band at 1627.28 cm^−1^ is due to the O-H bending of water molecules [44]. The FTIR spectrum range 2000 to 2300 cm^−1^ shows a weak bond corresponding C≡S and nitrile bond C≡N [45]. Furthermore, N-H, O-H, C-H and thiourea bonds vibrated in the region 3350 to 3500 cm^−1^ [46]. The characteristic vibration symmetry of CTS NPs in the FTIR study is in accordance to the XRD results.

### 3.2. X-ray Photoelectron Spectroscopy

**Figure 3:**
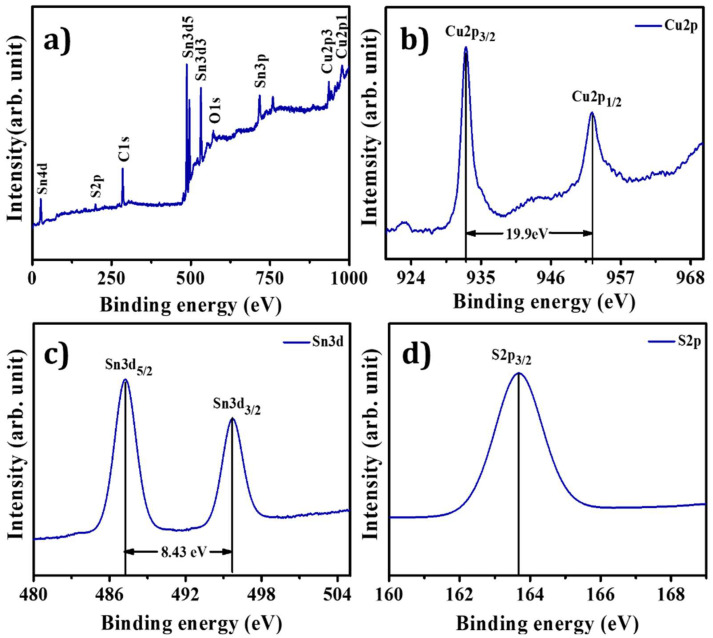
(**a**) Total XPS survey spectrum, (**b**) copper XPS survey spectrum, (**c**) tin XPS survey spectrum, and (**d**) sulfur XPS survey spectrum of CTS NPs.

X-ray photoelectron spectroscopy investigated the oxidation states and bonding changes in CTS NPs (see Figure 3a–d). The XPS spectra highlight the occurrence of Cu, Sn, S, O, and C elements. The occurrence of carbon and oxygen could be possible due to the environment (see Figure 3a). Figure 3b shows peaks located at 932.60 and 952.50 eV having binding energy splitting of 19.9 eV, highlighting the presence of Cu^1+^ state. Figure 3c shows Sn 3d peaks, which occurred at 487.26 and 495.69 eV, with its peak separation of 8.43 eV confirming the Sn^4+^ state. The presence of sulfide species confirmed by the sulfur 2p_3/2_ peak occurred at 163 eV, which agrees with the presence of sulfide species in the S^2−^ state (see Figure 3d). Thus, these results match reported data of CTS material in the literature [47,48,49,50].

### 3.3. Scanning Electron Microscopy (SEM)

**Figure 4:**
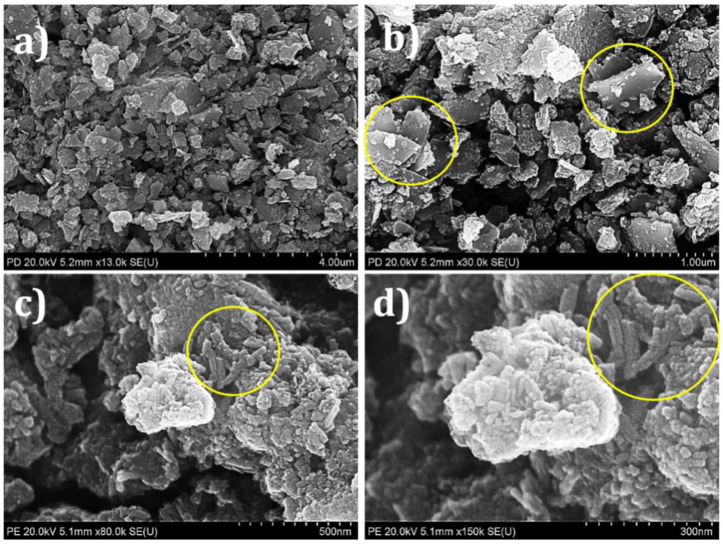
SEM micrographs of CTS NPs at various magnifications i.e., (**a**) 4.0 µm, (**b**) 1.0 µm with yellow circles indicating nanoparticles formation, (**c**) 500 nm with yellow circle indicating spherical nanostructures, and (**d**) 300 nm with yellow circle indicating agglomeration of oval-like spherical-shaped NPs with white spongy clusters.

The surface morphology of semiconducting material is significant in optoelectronic applications. Figure 4a–d demonstrates SEM images of CTS NPs at various magnifications. This result reveals that CTS powder is an agglomeration of different NPs. At the magnification of ×13,000, CTS NPs show that they are assembled and forming irregular agglomerates (see Figure 4a). At a magnification of ×30,000, the outer surface of the CTS material is constructed by the aggregation of NPs. Figure 4b illustrates the nanoplate-like morphology indicated by the yellow circle. Figure 4a,b exhibits the fabrication of a mixture of tiny clusters of NPs with a horizontally aligned plate-like structure. These plates are separated from each other and create voids between them. Additionally, these clusters of NPs and leaves consist of a numerous mesopore surface. It is clear from the micrograph of Figure 4c that the particles are smaller, irregular, and have a 2D structure, revealing uniform oval-like spherical-shaped NPs, as indicated by the green circle at a magnification of ×80,000. The grain sizes are found to be in the 500 nm range. At a magnification of ×150 k, CTS shows agglomeration of oval-like spherical-shaped NPs with white spongy clusters (see Figure 4d). The agglomeration was mainly due to high surface energy with a smaller size [51]. In addition to these particles, crystallites and randomly oriented particles can be seen. Microscopy magnification has revealed that the agglomeration of more minor constituents forms almost all particles. The result detected in the existing work is analogous to the effects described previously for CTS nanostructures achieved through the solvothermal route [52]. In general, CTS NPs form with mesopores surface morphology which is suitable for dye degradation.

### 3.4. Brunauer–Emmett–Teller (BET) Analysis

**Figure 5:**
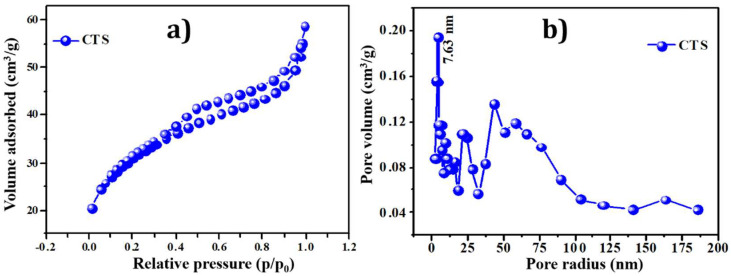
(**a**) N2 adsorption-desorption isotherm curve and (**b**) pore size distribution of CTS NPs.

The photocatalytic dye degradation performance of the active electrode material is mainly dependent on the surface conditions. The surface area plays an essential role in dye degradation for light absorption at different incident angles. The specific surface area of CTS NPs prepared at 180 °C hydrothermal temperature is measured by BET analysis. The higher specific surface area and appropriate pore volume of prepared NPs are essential for getting the best electrochemical dye degradation performance [5]. As the high specific surface area provides the more significant electroactive sites for electrochemical reactions, and appropriate pore volume offers the easiest way for an intercalation/deintercalation process. Figure 5a provides the N2 adsorption–desorption isotherms measured for the CTS powder sample in the relative pressure (ρ/ρ0) range of 0.0–1.0. The curve shows the type IV isotherm attended by the H3 type hysteresis loop. This indicated the existence of mesopores in the sample, with the pore diameter ranging from 2 to 50 nm [53]. The analysis shows the presence of mesopores with specific surface areas of 87.31 m^2^ g^−1^ of CTS material. Meanwhile, the pore size was calculated using the BJH method, and the result was 7.63 nm. The surface area and pore size are positively related to photocatalytic activity. Therefore, the photocatalytic activity of CTS NPs was higher [5]. Figure 5b shows the Barrett–Joyner–Halenda (BJH) pore size distribution plot of CTS NPs. The maximum pore size distribution occurs within 2 to 50 nm. The results from BET analysis support the SEM morphology as the nanoplates-like CTS possess maximum surface area.

### 3.5. Diffuse Reflectance Spectroscopy (DRS) Analysis

**Figure 6:**
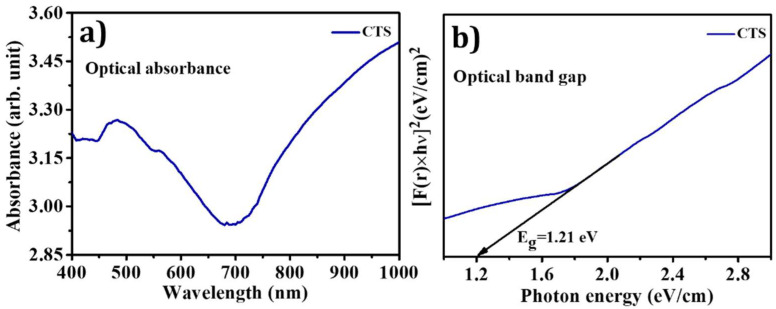
(**a**) Optical absorption spectrum and (**b**) optical band gap plot of CTS NPs.

One of the essential characteristics of a promising photocatalyst is its optical properties. The band-gap is a significant factor of semiconductor materials, which decides the definite generation of carriers [54]. The UV-Visible diffuse reflectance absorption spectrum (DRS) of CTS NPs is illustrated in Figure 6a. The absorption spectrum of CTS NPs showed the characteristic band-gap absorption edge at 700 nm. This spectrum indicates that CTS NPs absorb the entire visible section of electromagnetic waves and that the tail is prolonged to a lengthier wavelength. This circumstance reflects the superior morphology of this sample, which allows for the scattering of incident light in the interior of molecules. This increases the photon path length and causes the reflection of incident light to shrink, a phenomenon known as the light-trapping effect [55]. The band-gap of CTS NPs was determined using the Kubelka–Munk (K–M) standard equation given below:

(1) $${\left[F\left(r\right)h\nu \right]}^{\frac{1}{n}}=A\left(E_{g}-h\nu \right)$$

where *F*(*r*) is the Kubelka–Munk (K–M) function (i.e., ks), *s* is the scattering factor (s=2R), *k* is the molar absorption coefficient (i.e., $k={\left(1-R\right)}^{2}$), *R* is the reflectance data from DRS analysis, hν is the photon energy, and n are different values for allowed direct transition, $n=\frac{1}{2}$ and allowed indirect transition n=2. Equation (4) is similar to the Tauc plot. The band-gap plot of ${\left[F\left(r\right)h\nu \right]}^{2}$ verses hν for the CTS sample calculated from DRS using the K–M function is displayed in Figure 6b. The nature of the plot shows a direct interband transition. The extrapolated straight line depicts the material’s band-gap. The obtained band gap value of CTS NPs (1.20 eV) was matched with reported values of CZTS in the literature [54].

### 3.6. Photocatalytic Activity

**Figure 7:**
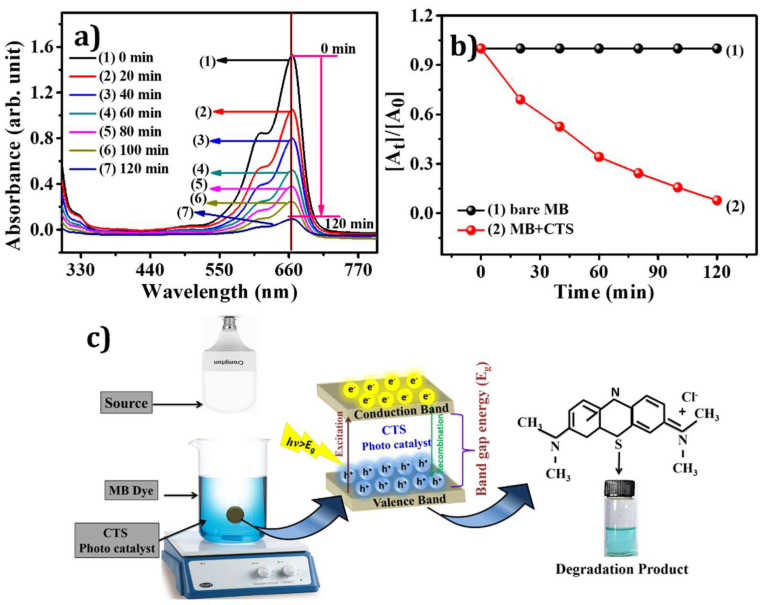
(**a**) UV-Vis absorption spectra of MB degradation with CTS as a catalyst, (**b**) plot of [*A_t_*]/[*A*_0_] as a function of radiation time for CTS nanoparticles, and (**c**) schematic representation of the possible photocatalytic degradation mechanism under visible-light irradiation with CTS as a catalyst.

The absorbance spectra of a water-based dye solution of MB in the presence of 25 mg CTS NPs under visible light irradiation are shown in Figure 7a. The peak at 664 nm is taken as the reference peak of the MB dye solution. The degradation of MB dye does not occur in the absence of a photocatalyst. Under visible light irradiation, however, the dye solution exhibits exponential degeneration in the characteristic peak in the presence of photocatalyst (CTS NPs). As time surges, the decolorization of the dye within the solution occurs; as revealed in Figure 7a, the readings of the absorbance spectrum were obtained at 20 min of rest. Later, Figure 7a clearly shows that the peak of the MB dye solution nearly disappeared after 120 min, and the dye solution became colorless. According to Figure 7a, there is a 95% decline within 120 min of the dye solution’s absorbance peak. The degradation rate of the reaction was calculated using the following equation:

(2) $$\mathrm{The}\;\mathrm{efficiency}\;\mathrm{of}\;\mathrm{degradation}\;\left(\%\right)=\left[\frac{\left(A_{0}-A_{t}\right)}{A_{0}}\right]\times 100$$

where *A*_0_ is the initial absorbance of MB dye in the absence of a catalyst and *A_t_* is the absorbance of the dye solution at time t in the presence of a catalyst. Figure 7b denotes the degradation rate of MB dye solution in the presence and absence of CTS NPs catalyst. Under the illumination of visible light, a tiny quantity of CTS NPs shows noble adsorption and degradation, the same as in the report [56]. Figure 7b shows rate constant *k_app_* was 0.0021 min^−1^.

(3) $$\ln\left(\frac{A_{t}}{A_{0}}\right)=-k_{app}t$$

Similarly, the value of ‘*k*’ was found to be 0.0848 min^−1^ g^−1^ using Equation (4).

(4) $$k=\frac{k_{app}}{m}$$

where ‘*m*’ is the mass of the catalyst. CTS NPs’ degradation effect concludes that they can be used in wastewater treatment in textile and other industries.

Figure 7c depicts a schematic representation of photocatalytic degradation of CTS NPs under visible-light illumination. As a result, CTS has p-type semi-conductivity, which means that holes are the primary carriers, advantageous for oxidizing organic compounds. Under visible light illumination, electrons (*e*^−^) are excited from the valence band (VB) to the conduction band (CB) of CTS NPs, resulting in the production of holes (*h*^+^) in the VB [57,58]. CTS NPs are stable in the environment; metal ion discharge does not contribute much to the photocatalytic degradation mechanism. In contrast, under visible-light irradiation, due to the defects side of CTS NPs or visible light electron-hole pair, the process of UV activating the production of reactive oxygen species (ROS) occurs. The generation of ROS is mainly because of electron–hole pairs and these ROSs, such as hydroxyl radicals $\left(OH^{•}\right)$ and superoxide radical anions $\left(O_{2}^{•-}\right)$. The $OH^{•}$ radicals are the primary active species in the photocatalytic degradation of organic pollutants. Holes and hydroxyl radicals can oxidize MB into degradation products [57]. The comparative elevation of degradation in CTS NPs shows high photon absorption in the visible region and the absence of any binary phases; however, the presence of binary phases in the NPs causes a non-radiative permutation of carriers, reducing their efficacy. Figure 7c proves that electron–hole pairs’ production depends on the interaction of CTS NPs with light. Manufactured ROS interacts with organic matter (MB) and degrades them into reduced harmful yields [37]. The various photocatalyst studies for the photocatalytic degradation of the MB dye are summarized in Table 1. The probable reaction phases are as follows:

(5) $$\mathrm{Step}\;1:\;h\nu +Catalyst\left(CTS\right)\to electron\left(e^{-}\right)+hole\left(h^{+}\right)$$

(6) $$\mathrm{Step}\;2:\;h^{+}+H_{2}O\to OH^{•}+H^{+}$$

(7) $$\mathrm{Step}\;3:\;e^{-}+O_{2}\to O_{2}^{•-}$$

(8) $$\mathrm{Step}\;4:\;O_{2}^{•-}+H_{2}O_{2}\to OH^{•}+OH^{-}+O_{2}$$

(9) $$\mathrm{Step}\;5:\;OH^{•}+\mathrm{MB}\;\mathrm{molecules}\to \mathrm{degraded}\;\mathrm{product}$$

### 3.7. Antimicrobial Activity

**Figure 8:**
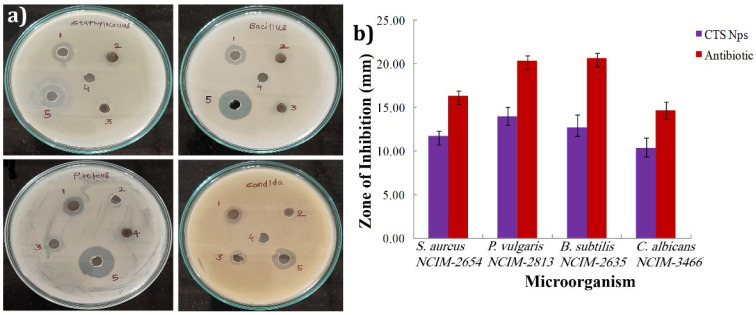
(**a**) Zone of inhibition: (1) CTS NPs + DMSO, (2) DMSO, (3) CTS + H_2_O, (4) H_2_O, and (5) antibiotic. (**b**) The statistical analysis of CTS NPs against tested microbial and fungal organisms.

The water rectification process contains organic pollutants and many other contaminants available in the wastewater. These toxin materials have a fragment of microbes, and it was reported earlier that the growth of pathogenic bacteria in the water is mainly because of organic metals [68]. Therefore, it is found that the photocatalyst has the property of inhabitation ability of microbial contaminants [69]. Commercial antibiotic (50 μg/mL) and antibacterial activity of CTS NPs (50 μg/mL) were scrutinized, and a significant zone of inhibition was detected. Figure 8a demonstrates a photograph representing the antibacterial activity of CTS NPs against three bacterial strains, *S. aureus* (*NCIM-2654*) gram-positive, *P. vulgaris (NCIM 2813)*, and *B. subtilis* (*NCIM-2635*) gram-negative, using streptomycin as a positive control and dimethylsulfoxide (DMSO) and distilled water as a negative control. Similarly, one fungal strain, *C. albicans* (*NCIM-3466*), was treated with fluconazole as the standard drug after incubation for 12 h. The antimicrobial potential of CTS NPs was studied using the agar gel diffusion method. Suspensions of CTS NPs drops were applied on round filter paper in a disc. Preparation of the DMSO medium was achieved by using the liquid auger suspension. The bacterial strains, i.e., *S. aureus*, *P. vulgaris*, *B. subtilis*, and one fungal strain, i.e., *C. albicans*, were prepared separately for each sample and control. The dilution of the CTS NPs sample was conducted as 2.5 mg/mL, and 10 μL drops of the sample were mixed in bacterial and fungal suspensions. The incubation of three bacterial and one fungal control suspensions was at 37 °C for 12 h. It can be deduced from the present study that CTS NPs have an excellent antimicrobial activity toward the three bacterial strains. *S. aureus*, *P. vulgaris*, *B. subtilis* and one fungal strain *C. albicans*. From the zone of inhibition (ZOI) values, CTS NPs were more efficient in inhibiting the growth of the bacterial strain *P. vulgaris*, as the maximum ZOI was 14.00 ± 1.00 mm. The ZOIs of CTS NPs against *S. aureus* and *B. subtilis* bacterial strains were 11.67 ± 0.58 mm and 12.67 ± 1.15 mm, respectively, as illustrated in Table 2. Similarly, the ZOI against the fungal strain C. albicans was 10.33 ± 1.53 mm.

These results were compared to the commercial antibiotic streptomycin, which is only applicable to bacterial strains. The ZOIs were 16.33 ± 0.58 mm, 20.67 ± 0.58 mm, and 20.33 ± 0.58 mm for *S. aureus, P. vulgaris,* and *B. subtilis*, respectively (from Figure 8a) [70]. Similarly, the ZOI of one fungal strain, *C. albicans*, using fluconazole as the standard drug, was 14.67 ± 1.00 mm, which is more efficient. The above data represent the mean ± standard error of three replicates illustrated in Table 2. Figure 8b illustrates the statistical analysis of CTS NPs antibiotics against bacterial and fungal strains. Additionally, DMSO + CTS NPs show good ZOIs compared to bare DMSO, H_2_O + CTS NPs, and bare H_2_O, as shown in Figure 8a. CTS possesses p-type characteristics; meanwhile, it resides in holes as the majority carriers. Thus, it degraded an enormous percentage of *P. vulgaris* as it collated with *S. aureus* and *B. subtilis*. Bacterial degradation is commonly connected with oxidative stress caused by ROS generated by functional materials. The cell membrane is the most crucial fragment of bacteria which safeguards it from the severity of the environment. The damage of this membrane in bacteria can lead to physiochemical changes resulting in inactivation. This stress leads to polyunsaturated acids and crust lipids destruction leading to free radicals that break the bonds. Later, as a second toxic reactive messenger, aldehydes destroy internal protein molecules and cause leakage of the membrane, which inactivates bacteria [71]. 

## 4. Conclusions

In conclusion, Cu_2_SnS_3_ (CTS) NPs were synthesized using a simple and environmentally friendly hydrothermal technique. The XRD study shows that CTS NPs exhibit a tetragonal structure with a kesterite phase. FTIR and XPS studies confirmed the formation of CTS compounds with pure phases. The morphological study revealed the formation of oval-like spherical-shaped NPs with white spongy clusters, consisting of several small crystallites that exist due to aggregation of the NPs. Additionally, the CTS sample’s band-gap value was 1.20 eV, leading to superior photocatalytic activities.

Moreover, CTS NPs exhibit high efficiency for MB dye degradation, thus making them a potential candidate for dye treatment in wastewater. There is a ~95% decrease in the absorbance peak of the dye solution within 120 min. The antimicrobial results demonstrated that CTS NPs display excellent antimicrobial and antifungal activity against three bacterial strains, i.e., *S. aureus*, *P. vulgaris*, *B. subtilis*, and one fungal strain *C. albicans*. Hence, it is proven that the CTS NPs are photocatalytically and antimicrobially active when exposed to visible light. As a result, the current study reports a new approach to acquiring NPs with a potential for dye degradation, UV protection, and antifungal and antibacterial areas.

## Figures and Tables

**Table 1:** Summarizes the different photocatalyst studies for the photocatalytic degradation of the MB dye.

Photocatalyst	The Initial Concentration of MB Dye (mg/L)	Light Source	Degradation %	Irradiation Time (min)	Ref. No.
CTS	25	Visible light	95	120	Proposed
CTS	25	Visible light	90	150	[37]
CZTS	15	Visible light	94	120	[59]
TiO_2_	10	Sunlight	96	150	[60]
ZnO	10	Visible light	99	120	[61]
CdS	20	Visible light	92	100	[62]
Ag_3_PO_4_	2	Visible light	99	60	[63]
Fe_3_O_4_	12	UV light	74	60	[64]
In_2_O_3_	10	UV light	98	40	[65]
CuO	5	Visible light	93	40	[66]
WO_3_	5	Visible light	94	90	[67]

**Table 2:** The zone of inhibition of three bacterial strains. *S. aureus, B. subtilis, P. vulgaris* and one fungal strain, *C. albicans*.

Entry	Antibacterial Activity			Antifungal Activity
	*S. aureus (NCIM-2654)*	*B. subtilis (NCIM-2635)*	*P. vulgaris (NCIM 2813)*	*C. albicans (NCIM-3466)*
CTS NPs	11.67 ± 0.58	12.67 ± 1.15	14.00 ± 1.00	10.33 ± 1.53
Streptomycin	16.33 ± 0.58	20.67 ± 0.58	20.33 ± 0.58	-
Fluconazole	-	-	-	14.67 ± 1.00

## Data Availability

Not applicable.
